# Hepatoprotective and antioxidant activity of hydroalcoholic extract of *Stachys pilifera. Benth* on acetaminophen-induced liver toxicity in male rats

**DOI:** 10.1016/j.heliyon.2019.e03029

**Published:** 2019-12-24

**Authors:** Mahboubeh Mansourian, Ali Mirzaei, Nahid Azarmehr, Hossein Vakilpour, Esmaeel Panahi Kokhdan, Amir Hossein Doustimotlagh

**Affiliations:** a Medicinal Plants Research Center, Yasuj University of Medical Sciences, Yasuj, Iran; b Department of Pharmacology, Faculty of Medicine, Yasuj University of Medical Sciences, Yasuj, Iran; c Student Research Committee, Yasuj University of Medical Sciences, Yasuj, Iran; d Department of Clinical Biochemistry, Faculty of Medicine, Yasuj University of Medical Sciences, Yasuj, Iran

**Keywords:** Food science, Animal science, Biological sciences, Health sciences, Hepatobiliary system, Pharmacology, Toxicology, Acetaminophen, Hepatotoxicity, Oxidative stress, *Stachys pilifera. Benth*

## Abstract

**Background:**

Acetaminophen (APAP) at high doses causes adverse side effects such as hepatotoxicity. The aim of the current study was to investigate the hepatoprotective and antioxidant effects of hydroalcoholic extract of *Stachys pilifera. Benth* (SP) on hepatotoxicity induced by APAP in male rats.

**Methods:**

Adult male Wistar rats were allocated into four groups: control (C), APAP (2 g/kg), APAP + SP (500 mg/kg), and APAP + Silymarin (SM, 100 mg/kg) as positive control group. On the seventh day, the rats were sacrificed after taking blood samples. Then levels of biochemical parameters, oxidative stress markers and activity of antioxidant enzymes were measured.

**Results:**

In the APAP group, aspartate aminotransferase (AST) and alanine aminotransferase (ALT) enzymes activity was significantly increased and the level of protein carbonyl (PCO) was insignificantly increased as compared to control group. In addition, the activity of glutathione peroxidase (GPX) and total thiol in the APAP group was significantly decreased compared to the normal rats. *Stachys pilifera. Benth* extract administration significantly reduced the activity of AST and ALT enzymes and the level of PCO compared to the APAP group, while significantly elevated the activity of GPX enzyme.

**Conclusion:**

Hydroalcoholic extract of SP diminishes hepatotoxicity induced by APAP by reducing the amount of liver function indicators (AST and ALT). Furthermore, the hydroalcoholic extract of SP is capable of reducing oxidative stress through inhibiting protein oxidation as well as boosting the activity of GPX enzyme. In this respect, the hepatoprotective impact induced by the SP extract may possibly be attributable to its reactive oxygen species scavenging and antioxidant properties.

## 1 Introduction

Acetaminophen (APAP- N-Acetyl-p-Aminophenol) is one of the most universally recommended and consumed pain-relievers, reduces fever in nursing care. It is a beneficial and potent drug in common doses in spite of having hepatotoxic effects. The APAP is not a problem in adults up to 4 g/day; however, some studies have shown that APAP can have harmful effects even in therapeutic doses in susceptible individuals, including alcoholic patients (Larson, 2007). The hepatotoxic effect of this drug may cause acute liver failure. In some cases, liver transplantation is the only treatment option to survive the patient. It should be noted that 90% of APAP is metabolized by glucuronidation and sulfation to non-toxic metabolites and less than 10% of APAP is metabolized to NAPQI (N-acetyl-p-benzoquinone imine) via cytochrome P450 (Tezcan et al., 2018). Then, the NAPQI (responsible for the hepatotoxicity of APAP) reacts with glutathione and converts it to various non-reactive molecules. After a toxic dose of APAP, about ninety percent of hepatic total glutathione is depleted and the hepatotoxicity occurs. In hepatotoxicity induced by APAP, the activity level of aspartate aminotransferase (AST) and alanine aminotransferase (ALT) enzymes increases 24 h after drug administration (Aycan et al., 2014). In excessive use of APAP, the NAPQI causes an imbalance between the production and removal of free radicals and leading to oxidative stress (Tezcan et al., 2018; Jaeschke, 2015). The factors progressing chronic liver disease are free radical-induced oxidative stress and chronic inflammation resulting from the release of proinflammatory cytokines from the liver Kupffer cells (López-Reyes et al., 2008). The enzymatic and non-enzymatic defense systems of the normal hepatocytes protect them against free radicals and reactants; the enzymatic defense system includes catalase (CAT), superoxide dismutase (SOD) and glutathione peroxidase (GPX) which rapidly react with and remove reactive oxygen species (ROSs) such as superoxide, hydrogen peroxide and hydroxyl radical (Mansourian et al., 2018; Lee et al., 2004). The cell can tolerate mild oxidative stress; but in a more severe state, oxidative compounds or ROS react with cellular constituents such as DNA, lipids, proteins and cell membrane, and subsequently cause pathological problems (Geetha et al., 2007). One of the most common changes in protein oxidation is the protein carbonyl (PCO) formation (Bayr, 2005), which is used as a marker for the development of ROS-induced changes in proteins (Iwai et al., 2010).

*Stachys pilifera. Benth* (SP) belonging to the Lamiaceae family and the *Stachys* genus grows in tropical and subtropical areas. This genus has 34 species in Iran, 13 of which are native. Phytochemical studies have shown the presence of compounds such as flavonoids, phenylethanoid glycosides, diterpenes, saponins, terpenoides and steroids in *Stachys* species (Panahi Kokhdan et al., 2017; Sadeghi et al., 2014; Shaheen et al., 2017; Ebrahimabadi et al., 2010). Previous studies have shown that the plant has cytotoxic, antioxidant, antibacterial (Farjam et al., 2011), anti-tumor (Jassbi et al., 2014; Farjam et al., 2011), anti-inflammatory (Hajhashemi et al., 2007; Sadeghi et al., 2014), hepatoprotective (Panahi Kokhdan et al., 2017) and immunoregulatory effects (Amirghofran et al., 2007). Aerial parts of the plant are used as herbal teas in various conditions, such as infections, asthma and rheumatoid arthritis (Sadeghi et al., 2014). The aim of the present study was to examine the hepatoprotective and antioxidant properties of hydroalcoholic extract of SP on hepatotoxicity induced by APAP.

## 2 Materials and methods

### 2.1 Extraction

Aerial parts of SP, including stems and leaves were collected in spring 2018 from the suburbs of Yasuj, Iran. The plant was authenticated by Dr. A. Jafari from Department of Botany, Center for Research in Natural Resource and Animal Husbandry, Yasuj University, Yasuj, Iran, where a voucher specimen (herbarium No. 1897) was deposited. After collecting the SP plant samples, they were cleaned and kept away from direct light in the room air for several days to dry, crushed and ready to be extracted. Thus, 100 g of dried plant was soaked with 1000 mL of solvent (70% ethanol). The resulting mixture was placed at 37 °C for 48 h, then filtered with Whatman Filter Paper No.1 and collected as much as possible by rotary evaporator under vacuum conditions. Next, the extract was dried at 50 °C and stored in a freezer at -20 °C (Nadimi et al., 2013).

### 2.2 Chemicals

Acetaminophen (APAP), ethylenediaminetetraacetic acid (EDTA), 5,5′-dithiols-2-nitrobenzoic acid (DTNB), thiobarbituric acid (TBA), and acetonitrile were obtained from Sigma Chemical Co (St Louis, MO, USA). 2, 4-dinitrophenylhydrazine (DNPH) and Trichloroacetic acid (TCA) were obtained from Merck (Germany). All materials used were of analytical purity.

### 2.3 Animals and experimental conditions

The experimental procedures of this study were confirmed by the Ethics Committee of Yasuj University of Medical Sciences (Yasuj, Iran) and then the rats were controlled according to “Guide for Laboratory Animal Care” (NIH Publication No. 86-23). In this study, 24 adult male Wistar rats (225 ± 25 g) were kept in an environment under a 12:12 h light/dark cycle with well-ordered temperature (24 ± 2 °C) and humidity (55–60%). All rats had *ad libitum* access to water and normal diet to drink and fed. After being adapted to the laboratory environment for one week, animals were randomly allocated into 4 groups of 6 as follows:

**Control (C):** distilled water (p.o.) was administered to rats for a period of 7 days; **Acetaminophen (APAP):** distilled water (p.o.) was administered to rats for a period of 7 days; a single injection of APAP (2 g/kg; p.o.) was given on the 6^th^ day (Azarmehr et al., 2019); **APAP + SP extract:** hydrochloric extract of SP (500 mg/kg; p.o.) (Panahi Kokhdan et al., 2017) was administered to rats for a period of 7 days; a single injection of APAP (2 g/kg; p.o.) was given on the 6^th^ day; **Positive control group: APAP + Silymarin (SM):** SM (100 mg/kg; p.o.) was administered to rats for a period of 7 days; a single injection of APAP (2 g/kg; p.o.) was given on the 6^th^ day.

On the 7^th^ day, blood was taken from rats. After sacrificing the rats, part of the liver tissue was homogenized with a homogenizer in a potassium phosphate buffer (pH 7.4 and 10 mM) and stored at -20 °C. The blood samples were collected in heparin-containing tubes, plasma was prepared and enzymes activity including ALT and AST were measured. Measurement of oxidative stress markers such as total thiol and PCO were also determined. In addition, the activity of antioxidant enzymes (including CAT, GPX and SOD) were measured in liver tissue homogenates.

### 2.4 Biochemical analysis

The activity level of liver enzymes (including AST and ALT) were measured using the Pars Azmoon kit and calorimetric method.

### 2.5 Oxidative stress markers

#### 2.5.1 Determination of protein carbonyl (PCO) content

The tissue homogenates and plasma were incubated for 60 min in dark conditions at room temperature in the presence of DNPH reagent (10 mM) in 2 M HCl. The samples were added with 50% TCA and centrifuged; the precipitate was added with ethanol/ethyl acetate 1: 1 (v/v) solution. The precipitate from centrifugation was added with guanidine hydrochloride solution (6 M), and the samples were kept warm at 37 °C for 15 min. Finally, the optical density (OD) of supernatant was read at 370 nm and the PCO content was determined using a molar absorptivity of 2.2 × 10^4^ M^−1^cm^−1^ (Kholari et al., 2016).

#### 2.5.2 Determination of total thiol content

The total thiol content was determined based on the reaction of DTNB with thiol groups and the formation of 2-nitro-5-thiobenzoic acid and di-sulfide compounds. Briefly, 25 μL of the sample, 10 μL of DTNB (10 mM), 150 μL of Tris-EDTA and 790 μL of absolute methanol were added to a microtube. The microtube was then slowly mixed and incubated for 15 min at 25 °C. The OD of the supernatant read at a wavelength of 412 nm and the total thiol content was calculated using a molecular absorptivity of 13600 M^−1^cm^−1^ (Sadeghi et al., 2019).

#### 2.5.3 Measurement of the antioxidant enzymes

GPX, SOD and CAT enzymes activity was measured in the liver homogenates using ELISA kits (ZellBio GmbH, Ulm, Germany) according to the kit instructions.

#### 2.5.4 Statistical analysis

Data were evaluated by SPSS version 18 software using one-way analysis of variance (ANOVA) with LSD and Tukey's post hoc correction. The results were expressed as mean ± S.E.M. A P-value less than 0.05 was determined to be statistically significant.

## 3 Results

### 3.1 Biochemical parameters

**Figure 1:**
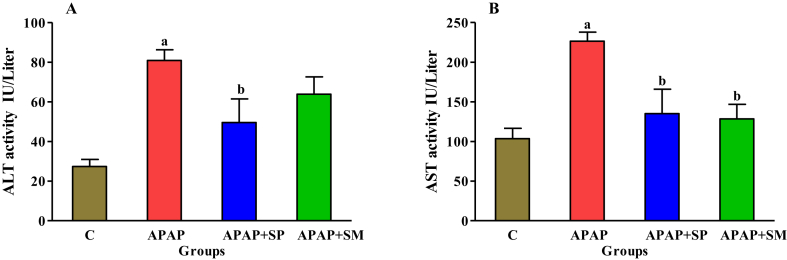
Effect of ethanolic extract of SP on the plasma level of ALT (A) and AST (B) in hepatotoxicity induced by APAP. C: control; APAP: acetaminophen; APAP + SP: acetaminophen and 500 mg/kg of ethanolic extract of *Stachys pilifera*; APAP + SM: acetaminophen and 100 mg/kg of silymarin. Each value represents mean ± SEM; ^a^P < 0 05 versus control group and ^b^P < 0 05 versus APAP group.

The plasma levels of the liver enzymes were determined to evaluate hepatotoxicity. According to Figure 1, the AST and ALT plasma levels had significantly augmented in the APAP group as compared those in the control group (p < 0.05), while the ethanolic extract of SP significantly decreased these markers (P < 0.05). Nevertheless, APAP + SM group as positive control group did not significantly reduce the plasma level of ALT compared to APAP group.

### 3.2 Oxidative stress markers

**Figure 2:**
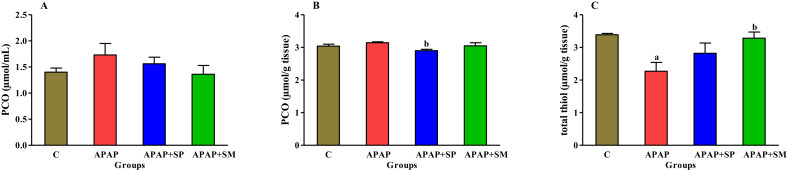
The impact of ethanolic extract of SP on levels of plasma PCO (A), liver PCO (B) and liver total thiol (C) in hepatotoxicity induced by APAP. C: control; APAP: acetaminophen; APAP + SP: acetaminophen and 500 mg/kg of ethanolic extract of *Stachys pilifera*; APAP + SM: acetaminophen and 100 mg/kg of silymarin; Protein carbonyl (PCO). Each value represents Mean ± SEM; ^a^P < 0 05 versus control group and ^b^P < 0 05 versus APAP group.

Our findings showed that the plasma PCO level in the APAP group was augmented by 23% as compared to the normal rats. The total thiol content was decreased by 49% compared to the normal group (P < 0.05). The PCO content (as the protein oxidation marker) in the liver homogenates of the APAP + SP extract group was significantly decreased compared to the APAP group (P < 0.05), but APAP + SM group as positive control group did not significantly change the PCO content in the hepatic homogenates compared to APAP group. The treatment of APAP-induced rats with hydroalcoholic extract of SP increased the total thiol content up to 24% in liver tissue homogenates (Figure 2). In addition, the SM restored the total thiol content in the APAP + SM group as compared to the only APAP group.

### 3.3 Activity of antioxidant enzymes

**Figure 3:**
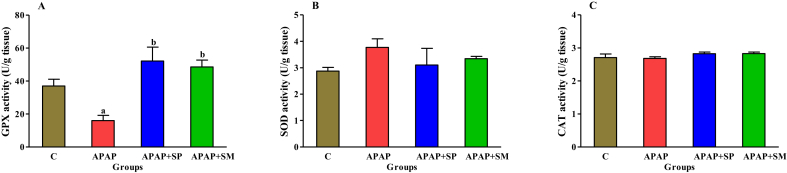
Effect of SP extract on the antioxidant enzymes activity of GPX (A), SOD (B) and CAT (C) in hepatotoxicity induced by APAP. C: control; APAP: acetaminophen; APAP + SP: acetaminophen and 500 mg/kg of ethanolic extract of *Stachys pilifera*; APAP + SM: acetaminophen and 100 mg/kg of Silymarin. ^a^P < 0 05 versus control group and ^b^P < 0 05 versus APAP group.

As presented in Figure 3, the GPX activity was significantly decreased in the APAP group (P < 0.05), while treatment with ethanolic extract of SP and SM caused an obvious increase in this enzyme (P < 0.05). However, the activity of the SOD and CAT enzymes in the APAP group did not significantly change compared to other groups.

## 4 Discussion

In the present study, the APAP-induced hepatotoxicity model was developed to examine the antioxidant and hepatoprotective effects of ethanolic extract of SP on hepatic injury. The hepatotoxicity induced by APAP is a reliable model for the study of hepatoprotective factors, which has been used in several studies in various intraperitoneal and oral doses for hepatotoxicity induction (Aycan et al., 2014; Lucarini et al., 2014; Yao et al., 2015). The ALT and AST are transaminase enzymes that play a crucial role in converting amino acids into keto acids. The AST is predominantly present in the mitochondria of hepatocytes, and the ALT is mainly in hepatocyte cytoplasm; an increase in the ALT level is usually associated with an increase in the AST level (Hanafy et al., 2016); however, the ALT is more specific for liver tissue, and therefore, a better marker for liver damage (Parmar et al., 2010). As it was observed, the ALT and AST levels were increased markedly in the APAP group rats compared with that in the normal rats. APAP damages to hepatocytes, reduces cell membrane integrity, and consequently increases cellular leakage of transaminase enzymes (Hanafy et al., 2016). The administration of the SP extract significantly reduced the levels of ALT and AST enzymes, which indicates liver tissue regeneration and hepatocyte restoration in the groups treated with extract. Nevertheless, APAP + SM group as positive control group did not significantly reduce the plasma level of ALT compared to APAP group. Therefore, the ethanolic extract of SP had better effectiveness rather than SM.

The NAPQI is an active metabolite derived from APAP, which covalently binds to lipid, protein and DNA macromolecules. Additionally, the NAPQI can react with glutathione in the liver and deplete the antioxidant capacity (Yao et al., 2015). Even though oxidative stress products might react with DNA, lipids, and proteins; the proteins are considered to be more at risk since they frequently act as enzymes in cells. Among various oxidative changes occurring on proteins, the PCO is probably the first indicator formed from the reaction of free radicals with proteins (Alou-El-Makarem et al., 2014). The PCO levels in liver and kidney tissue homogenates of rats orally receiving APAP (3 g/kg) were significantly increased compared to the control group (El-Shafey et al., 2015). In another study, the oral administration of APAP at a dose of 3 g/kg markedly raised the PCO content in the hepatic homogenates (Cristani et al., 2016). Our finding indicated that the PCO content in the APAP group with oral dose of 2 g/kg was slightly increased compared with that in the normal rats; this difference may be due to the lower dose of APAP used in our study. In the current study, the administration of ethanolic extract of SP (500 mg/kg) significantly diminished the PCO content in the hepatic homogenates compared with that in the APAP group, but APAP + SM group as positive control group did not significant change in the PCO content in the hepatic homogenates compared to APAP group. Therefore, the ethanolic extract of SP had better effectiveness rather than SM. Due to the high levels of flavonoids in the SP extract, this compound probably inhibits the protein oxidation process and thus plays its antioxidant role.

All the plasma thiol groups are related to proteins; these groups are oxidative sensitive indexes that play a biotic role in the defense system against ROS (Terzioglu et al., 2017). The total thiol contains the protein thiol (P-SH) and glutathione (GSH) groups; there is little difference between the levels of total thiol and P-SH due to the insignificant content of GSH (Hu, 1993). In accordance with earlier study, our study showed that the total thiol content of the liver tissue in APAP-induced rats was significantly reduced compared with the control group (Kabirifar et al., 2017). Salwe et al., in 2017 reported that the GSH amount in the liver tissue of the APAP-induced rats was significantly reduced compared with that in the control group (Hajhashemi et al., 2007). The reduction of total thiol groups in our study is probably due to the production of NAQPI; this active metabolite is produced in the liver from APAP and depletes thiol groups such as GSH. Administration of hydroalcoholic extract of SP (500 mg/kg) increased the total thiol content in the liver tissue homogenates by 24% compared to the APAP group; this increase may be due to high antioxidant properties and free radical scavenging activity.

The CAT, SOD, and GPX; as antioxidant enzymes, are able to protect cellular components against injuries induced by ROS. Aycan et al., in 2014 found that SOD and GPX activity significantly increased and decreased in APAP-induced rats, respectively, compared to the control group (Aycan et al., 2014). Other studies indicated that the APAP injection markedly diminished the activity of GPX, SOD, and CAT antioxidant enzymes (Hajhashemi et al., 2007; Aydogan et al., 2016). In the present study, the GPX activity was markedly decreased in the APAP group as compared the normal rats, while the SOD activity showed a slight increase. The main free radicals are superoxide anions, hydroxyl radical and H_2_O_2_ (Fukuhara and Kageyama, 2005). The SOD is the main enzyme of the antioxidant system in the reduction of superoxide radicals (Aycan et al., 2014), and the GPX is the main enzyme in the reduction of hydroxyl radical (Vieira et al., 2011). Reducing GPX activity after APAP administration may be due to increased hydroxyl radical formation. Our results showed that the administration of SM and hydroalcoholic extract of SP significantly increase the GPX activity. This study examined the effect of the ethanolic extract of SP on hepatotoxicity induced by APAP; although the exact mechanism of the effect of the SP extract on the activity of antioxidant enzymes is unclear, this extract through reducing the hydroxyl radical formation and increasing the GPX gene expression may regenerate the activity of this antioxidant enzyme.

## 5 Conclusions

The present study, as the first one in this field, suggested that the hydroalcoholic extract of SP could lower hepatotoxicity induced by APAP via moderating the levels of AST and ALT as liver function markers. Besides, the hydroalcoholic extract of SP could mitigate oxidative stress through inhibiting protein oxidation and improving the activity of GPX enzyme. In addition, the ethanolic extract of SP had better effectiveness rather than SM as positive control. The hepatoprotective impact of the extract of SP might be as a result of its free radical scavenging and antioxidant activities. The finding of the present study implied that the ethanolic extract of SP may be taken into account as a valuable substitute to adjust hepatotoxicity induced by APAP.

## Declarations

### Author contribution statement

A. Doustimotlagh: Conceived and designed the experiments; Analyzed and interpreted the data; Wrote the paper.

A. Mirzaei: Conceived and designed the experiments.

N. Azarmehr, H. Vakilpour and E. Kokhdan: Performed the experiments; Contributed reagents, materials, analysis tools or data.

M Mansourian: Analyzed and interpreted the data; Wrote the paper.

### Funding statement

This work was supported by Medicinal Plants Research Center affiliated to Vice Chancellery for research, 10.13039/501100007119Yasuj University of Medical Sciences, Yasuj, Iran (Grant No. 960393).

### Competing interest statement

The authors declare no conflict of interest.

### Additional information

No additional information is available for this paper.
